# Osteotomy as an Intraoperative Determinant of Early Postoperative Outcomes After Mandibular Third Molar Extraction: A Secondary Analysis of a Randomized Clinical Trial

**DOI:** 10.3390/jcm15103756

**Published:** 2026-05-13

**Authors:** Wojciech Niemczyk, Daniel Selahi, Marzena Dominiak, Kacper Chowaniec, Wiktor Zalasiński, Rafał Wiench, Jakub Hadzik

**Affiliations:** 1 Department of Dental Surgery, Faculty of Dentistry, Wroclaw Medical University, Krakowska 26, 50-425 Wroclaw, Poland; selahi.badania@gmail.com (D.S.); marzena.dominiak@umw.edu.pl (M.D.); jakub.hadzik@umw.edu.pl (J.H.); 2 MCIW, Wrocław Medical Innovation Center, Krakowska 26, 50-425 Wroclaw, Poland; 3 Department of Periodontal Diseases and Oral Mucosa Diseases, Faculty of Medical Sciences in Zabrze, Medical University of Silesia, 40-055 Katowice, Poland; rwiench@sum.edu.pl

**Keywords:** molar, third, tooth extraction, osteotomy, postoperative complications, edema, swelling, trismus

## Abstract

**Background/Objectives:** Surgical extraction of impacted mandibular third molars is frequently associated with postoperative morbidity, including swelling, trismus, and pain. However, the extent to which osteotomy contributes to these outcomes remains unclear. The aim of this study was to evaluate osteotomy as an intraoperative determinant of early postoperative morbidity following mandibular third molar extraction. **Methods:** This study represents a secondary analysis of data obtained from a randomized clinical trial. Patients undergoing surgical removal of impacted mandibular third molars were categorized according to whether osteotomy was required during the procedure. Postoperative outcomes included surgical duration, facial swelling (primary outcome) assessed by linear facial measurements, maximal mouth opening (trismus), postoperative pain intensity, and early soft tissue healing evaluated using the Early Healing Index. **Results:** Procedures involving osteotomy were associated with significantly longer surgical duration, as well as greater postoperative swelling and trismus during the early postoperative period. The most pronounced difference in swelling was observed along facial measurement line A on postoperative day 3. Multivariable analysis confirmed that osteotomy remained independently associated with increased postoperative swelling and trismus after adjustment for age, sex, and the original six-arm treatment allocation. In contrast, no statistically significant differences were found between the groups in postoperative pain intensity or early soft tissue healing. **Conclusions:** Osteotomy during mandibular third molar extraction is independently associated with increased early postoperative morbidity, particularly in terms of swelling and trismus. However, bone removal does not appear to negatively affect early soft tissue healing of the surgical site.

## 1. Introduction

Surgical removal of impacted mandibular third molars is one of the most frequently performed procedures in oral and maxillofacial surgery [1,2]. Despite being considered a routine intervention, it is commonly associated with postoperative morbidity, including pain, facial swelling, trismus, alveolar osteitis, infection, and other local complications that may impair postoperative recovery [3,4,5]. Although impacted third molars are primarily considered from a surgical perspective, the management of impacted mandibular third molars remains controversial, particularly regarding the prophylactic removal of asymptomatic teeth [6]. At the same time, extracted third molars may represent a valuable biological source of dental pulp stem cells for applications in regenerative medicine [7,8,9]. Preoperative imaging plays an important role in surgical planning, as panoramic radiography and cone-beam computed tomography (CBCT) may assist not only in anatomical assessment but also in predicting surgical difficulty and the potential need for bone removal [10]. In addition, CBCT enables the identification of anatomical variations, such as accessory or bifid mandibular canals, which may influence surgical planning and help reduce the risk of intraoperative and postoperative complications [11].

The severity of postoperative symptoms following third molar surgery is influenced by several factors, including surgical difficulty, operative time, extent of tissue manipulation, and the inflammatory response triggered by surgical trauma. Previous studies have demonstrated that postoperative pain, swelling, and trismus are primarily associated with the inflammatory response induced by surgical trauma to both hard and soft tissues [12,13]. However, postoperative pain intensity does not always correlate directly with the magnitude of surgical trauma, suggesting that additional physiological and neurogenic mechanisms may also contribute to postoperative discomfort [14,15].

One of the key intraoperative factors potentially contributing to postoperative morbidity is the need for osteotomy during tooth removal. Bone removal is frequently required to facilitate extraction of impacted mandibular third molars; however, it inevitably increases the extent of surgical trauma. Greater tissue injury may therefore lead to a stronger inflammatory response, which may subsequently result in increased swelling and trismus during the early postoperative period [16,17,18].

Several surgical, pharmacological, and adjunctive strategies have been proposed to reduce postoperative morbidity after third molar surgery, including minimally traumatic surgical techniques, different flap designs, piezoelectric osteotomy, anti-inflammatory medications, photobiomodulation, and autologous platelet concentrates [19,20]. In addition to pharmacological interventions, non-pharmacological approaches such as kinesiology taping and tissue adhesives have also been investigated as methods for reducing postoperative swelling and pain and improving early wound healing; however, their effects remain inconsistent and are unlikely to fully counteract the impact of surgical trauma associated with procedures such as osteotomy [21,22,23,24,25].

Despite the large number of studies investigating postoperative management after third molar surgery, the specific role of osteotomy as an independent intraoperative determinant of postoperative morbidity remains incompletely understood particularly in the context of other perioperative and postoperative modifiers of healing. Most studies have focused on pharmacological protocols or surgical techniques, whereas the direct contribution of bone removal as a source of surgical trauma has been less frequently analyzed.

The aim of the current hypothesis-driven secondary analysis was to evaluate whether the use of osteotomy during surgical extraction of impacted mandibular third molars is associated with early postoperative morbidity.

## 2. Materials and Methods

### 2.1. Study Design

The present study represents a hypothesis-driven secondary analysis of cohort data derived from a previously conducted randomized clinical trial evaluating biologically active modifiers of postoperative healing following mandibular third molar extraction [26]. The data set has also been analyzed including systemic and radiological factors [10,27]; however, the current analysis addresses a distinct research question focused on the role of osteotomy as an intraoperative determinant of postoperative morbidity.

No additional patient recruitment or modifications to the original study protocol were performed. The present investigation is therefore based exclusively on an existing clinical dataset and should be interpreted accordingly.

The primary objective of this analysis was to evaluate whether the intraoperative requirement for osteotomy is associated with early postoperative morbidity following surgical extraction of impacted mandibular third molars.

The original randomized clinical trial was conducted in accordance with the Declaration of Helsinki and Good Clinical Practice guidelines. Ethical approval was obtained from the Bioethics Committee of Wroclaw Medical University (approval no. KB-705/2019), and the trial was registered at ClinicalTrials.gov (identifier: NCT07324213). All participants provided written informed consent prior to inclusion.

In the parent randomized clinical trial, patients were allocated to six treatment groups: control, photobiomodulation (PBM), A-PRF+, CGF, A-PRF+ combined with PBM, and CGF combined with PBM. In the present secondary analysis, the original six-arm treatment allocation was included as a covariate to account for the potential influence of postoperative adjunctive therapies, while the primary exposure of interest was the intraoperatively determined requirement for osteotomy. Importantly, osteotomy was not randomized but determined intraoperatively according to surgical necessity and was observed across all treatment groups. It should therefore be considered a non-randomized exposure within the analyzed dataset.

### 2.2. Participants

A total of 135 patients were initially enrolled in the randomized clinical trial. Of these, 122 participants completed the follow-up period and were included in the final analysis. Key inclusion criteria comprised generally healthy adults requiring surgical extraction of impacted mandibular third molars, while exclusion criteria included systemic conditions affecting healing, active infections, and contraindications to oral surgery, as described in detail in the primary study [26]. The Pederson difficulty index was used as a radiological proxy of surgical difficulty, and all cases were classified according to this index into minimal, moderate, and high difficulty categories [10]. Eligibility was determined based on clinical and panoramic radiographic assessment [26].

### 2.3. Surgical Procedure

All surgical procedures were performed under standardized clinical conditions at a university dental surgery center. To minimize operator-related variability, all interventions were carried out by a single experienced oral surgeon using a consistent surgical protocol.

Local anesthesia was administered using 4% articaine with epinephrine (1:200,000) via inferior alveolar nerve block with buccal infiltration. Surgical access was obtained using a mucoperiosteal envelope flap without vertical releasing incisions.

When necessary, bone removal was performed using a small round bur under continuous irrigation. The extent of osteotomy was limited to the minimum required to facilitate atraumatic tooth removal, in accordance with a minimally invasive surgical approach. Internal tooth sectioning was performed when indicated using rotary instruments to allow controlled separation of the crown and root components.

Following extraction, the surgical site was irrigated, and primary wound closure was achieved using non-resorbable sutures.

Surgical duration was recorded for each procedure and defined as the time from initial incision to completion of wound closure.

Postoperative management was standardized for all participants and included administration of nimesulide (100 mg). No routine antibiotic prophylaxis was used.

The decision to perform osteotomy was made intraoperatively based on surgical necessity and was not randomized; therefore, it should be interpreted as a non-randomized exposure within the study design.

### 2.4. Osteotomy Assessment

For the purposes of the present analysis, surgical procedures were categorized according to whether osteotomy of the surrounding bone was required during mandibular third molar extraction.

Based on the operative records, procedures were divided into two groups: (1) osteotomy group, in which removal of surrounding bone was required to facilitate extraction, and (2) non-osteotomy group, in which the tooth could be removed without bone removal.

The decision to perform osteotomy was made intraoperatively by the surgeon according to clinical necessity and standard surgical principles and should therefore be interpreted as a marker of surgical complexity rather than a randomized or treatment-related factor within the study design.

### 2.5. Postoperative Outcome Assessment

Postoperative morbidity was evaluated using clinical parameters including facial swelling, trismus, pain intensity, and early soft tissue healing.

The primary outcome was postoperative swelling, selected as a clinical surrogate of the inflammatory response to surgical trauma. Secondary outcomes included trismus, pain intensity, and early soft tissue healing.

All outcomes were assessed during standardized follow-up visits on postoperative days 1, 3, and 7.

#### 2.5.1. Pain

Postoperative pain intensity was assessed using an 11-point numeric rating scale (NRS), where 0 indicated no pain and 10 represented the worst imaginable pain [28]. Pain assessment was performed during scheduled follow-up visits conducted on postoperative days 1, 3, and 7.

During each follow-up visit, patients were asked by the clinician to evaluate their current level of pain using the NRS scale. The reported scores were recorded in the study database and subsequently used for statistical analysis.

The numeric rating scale was selected as a validated and widely used method for the assessment of postoperative pain intensity in clinical studies involving oral surgical procedures.

#### 2.5.2. Swelling

Facial swelling was evaluated using standardized line measurements between predefined anatomic landmarks on the facial skin. Measurements were performed using a flexible measuring tape.

Three measurement lines were recorded:lateral canthus–gonion (line A), tragus–labial commissure (line B), and tragus–pogonion (line C). Baseline measurements were obtained before surgery, and the same measurements were repeated during scheduled follow-up visits on postoperative days 1, 3, and 7.

Postoperative swelling was calculated as the difference between postoperative and baseline measurements for each anatomical line. Positive values indicated an increase in soft tissue dimensions relative to baseline.

#### 2.5.3. Trismus

Trismus was assessed by measuring maximal interincisal mouth opening in millimeters (mm) before surgery and during scheduled postoperative follow-up visits on days 1, 3, and 7. Maximal mouth opening was defined as the distance between the incisal edges of the upper and lower central incisors.

The degree of postoperative trismus was calculated as the difference between baseline maximal mouth opening and postoperative measurements obtained at each follow-up visit.

#### 2.5.4. Soft Tissue Healing

Soft tissue healing was evaluated on postoperative day 7 using the Early Healing Index (EHI), a clinical scoring system used to assess wound closure and the condition of the surgical site [29]. The EHI scale ranges from 1 to 5, with lower scores indicating better soft tissue healing.

Detailed descriptions of the outcome assessment procedures have been reported previously in the original randomized clinical trial.

### 2.6. Statistical Analysis

Statistical analysis was performed using R software 4.5.1 (R Foundation for Statistical Computing, Vienna, Austria). Descriptive statistics were calculated for all variables. Continuous variables were expressed as medians and interquartile ranges (IQR) due to the non-normal distribution of most clinical parameters.

Pain intensity was analyzed using numeric rating scale (NRS) scores, while trismus was evaluated as the reduction in maximal mouth opening expressed in millimeters (mm) relative to baseline measurements. Postoperative swelling was calculated as the difference between postoperative and baseline linear facial measurements.

Differences between the osteotomy and non-osteotomy groups were analyzed using the Mann–Whitney U test for continuous variables and the chi-square test for categorical variables. The level of statistical significance was set at *p* < 0.05.

Due to the inherent variability of linear facial measurements, small negative values in swelling measurements occasionally occurred and were interpreted as measurement variability around baseline rather than true tissue contraction.

As osteotomy was not randomized and may reflect surgical difficulty, multivariable adjustment was performed to partially account for potential confounding.

Multivariable linear regression analyses were performed to assess the independent effect of osteotomy on postoperative swelling (line A, day 3) and trismus (day 3), adjusted for age, sex, and the original six-arm treatment allocation from the parent randomized clinical trial. The six-arm treatment allocation was included to account for the potential influence of postoperative adjunctive therapies, including photobiomodulation and autologous platelet concentrates. Regression coefficients (β) with 95% confidence intervals (CI) were calculated. Multivariable analyses were limited to the primary outcome and the most clinically relevant secondary outcome to avoid model overfitting.

## 3. Results

### 3.1. Study Population

A total of 135 patients were initially enrolled in the randomized clinical trial. Of these, 122 participants completed the follow-up period and were included in the present analysis.

Detailed baseline characteristics of the study population have been reported previously in the primary publication of the randomized clinical trial [1].

Among the analyzed surgical procedures, osteotomy was required in 73 cases (59.8%), whereas 49 procedures (40.2%) were performed without bone removal, indicating that bone removal was a common intraoperative requirement in the study population. The extracted mandibular third molars included 63 left teeth (tooth 38) and 59 right teeth (tooth 48). Detailed distribution of impaction patterns, including Pell and Gregory classification and their relationship to surgical parameters, has been previously reported for the same study cohort in a dedicated radiological analysis, and was therefore not repeated in the present study [10]. Briefly, vertical (*n* = 48) and mesioangular (*n* = 35) impactions were the most common, followed by horizontal (*n* = 22) and distoangular (*n* = 17).

The proportion of procedures requiring osteotomy varied across treatment groups (approximately 44–77%), likely reflecting differences in surgical difficulty. Osteotomy was observed in all treatment arms and was therefore analyzed as an independent intraoperative factor, while treatment allocation was included as a covariate in the multivariable models.

### 3.2. Surgical Duration According to Osteotomy

Surgical duration was analyzed according to whether osteotomy was required during the extraction procedure.

Procedures involving osteotomy were associated with significantly longer operative times compared with procedures performed without bone removal. The median surgical duration was 37 min (IQR: 30–45) in the osteotomy group and 21 min (IQR: 15–27) in the non-osteotomy group (*p* < 0.001).

Osteotomy was observed across all treatment groups of the parent randomized clinical trial, indicating that bone removal was not restricted to any specific intervention arm.

Detailed comparisons of surgical duration between the two groups are presented in Table 1.

### 3.3. Osteotomy and Postoperative Pain

Postoperative pain intensity was analyzed according to whether osteotomy was required during the extraction procedure.

Patients who underwent procedures involving osteotomy tended to report slightly higher pain scores during the postoperative period compared with patients treated without bone removal. The median pain score on postoperative day 1 was 1 (IQR: 0–3) in the osteotomy group and 1 (IQR: 0–2) in the non-osteotomy group (*p* = 0.138).

Similarly, on postoperative day 3 the median pain score was 1 (IQR: 0–3) in the osteotomy group and 1 (IQR: 0–2) in the non-osteotomy group (*p* = 0.138). On postoperative day 7, pain scores were 1 (IQR: 0–3) and 0 (IQR: 0–2) in the osteotomy and non-osteotomy groups, respectively (*p* = 0.055).

These findings indicate that the requirement for osteotomy was not associated with increased postoperative pain.

Detailed comparisons of postoperative pain intensity between the osteotomy and non-osteotomy groups are presented in Table 2.

### 3.4. Osteotomy and Postoperative Swelling

Postoperative swelling was evaluated as the change in facial measurements relative to baseline values obtained before surgery.

Procedures requiring osteotomy were associated with greater postoperative swelling. The difference between the groups was most pronounced on postoperative day 3. The median increase in swelling measured alongline A was 5 mm (IQR: 0–8) in the osteotomy group and 0 mm (IQR: −5–5) in the non-osteotomy group (*p* = 0.017).

No statistically significant differences in swelling measured alongline Awere observed on postoperative day 1 (*p* = 0.690) or day 7 (*p* = 0.422).

For swelling measured alongline B, no statistically significant differences were observed between the groups. On postoperative day 3, the *p*-value approached the threshold of statistical significance (*p* = 0.054).

Forline C, a statistically significant difference between the groups was observed on postoperative day 1 (*p* = 0.043), whereas no significant differences were detected on postoperative day 3 (*p* = 0.229) or day 7 (*p* = 0.372). Significantly greater postoperative swelling was observed in the osteotomy group, particularly on postoperative day 3.

Detailed comparisons of swelling measurements for all anatomical lines (A, B, and C) and postoperative time points are presented in Supplementary Table S1, whereas results for swelling measured alongline A are summarized in Table 3.

### 3.5. Osteotomy and Postoperative Trismus

Postoperative trismus was evaluated as the reduction in maximal mouth opening relative to baseline measurements obtained before surgery.

Patients requiring osteotomy demonstrated significantly greater postoperative trismus compared with those treated without bone removal. On postoperative day 1, the median reduction in mouth opening was 13 mm (IQR: 5–21) in the osteotomy group and 6 mm (IQR: 2–18) in the non-osteotomy group (*p* = 0.025).

A similar pattern was observed on postoperative day 3, when the median reduction in mouth opening was 13 mm (IQR: 7–20) in the osteotomy group and 7 mm (IQR: 2–16) in the non-osteotomy group (*p* = 0.004). Differences between the groups remained significant on postoperative day 7, with median reductions of 6 mm (IQR: 2–14) and 2 mm (IQR: 0–8), respectively (*p* = 0.001). Detailed comparisons of postoperative trismus measurements are presented in Table 4. 

In a multivariable linear regression model adjusted for age, sex, and the original six-arm treatment allocation from the parent randomized clinical trial, osteotomy remained significantly associated with increased postoperative swelling on day 3 (line A) (β = 3.35 mm, 95% CI: 0.75–5.95, *p* = 0.012) as summarized in Table 5.

Multivariable analysis confirmed that osteotomy was independently associated with greater postoperative trismus on day 3 after adjustment for age, sex, and the original six-arm treatment allocation (β = 4.95 mm, 95% CI: 1.29–8.62, *p* = 0.008), as shown in Table 5. Male sex was associated with lower trismus values in the adjusted model (β = −5.45 mm, 95% CI: −9.27 to −1.63, *p* = 0.005).

As a sensitivity analysis based on available clinical variables, additional models including osteotomy and sex yielded consistent results. Osteotomy remained significantly associated with increased swelling on postoperative day 3 along line A (β = 3.37 mm, 95% CI: 0.80–5.94, *p* = 0.011) and greater trismus on postoperative day 3 (β = 5.26 mm, 95% CI: 1.77–8.75, *p* = 0.003).

**Figure 1:**
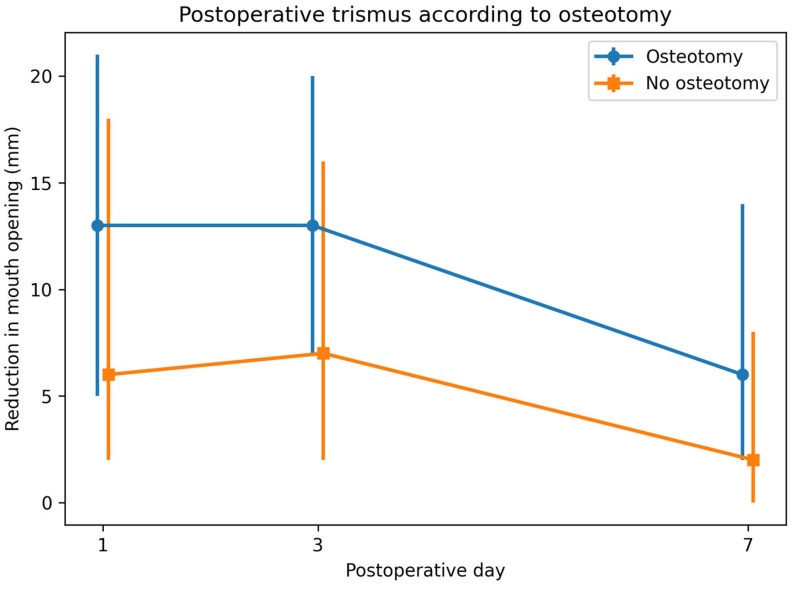
Postoperative trismus (reduction in maximal mouth opening relative to baseline) according to osteotomy on postoperative days 1, 3, and 7. Values represent median reductions in mouth opening.

Postoperative trismus was greater in the osteotomy group than in the no-osteotomy group on postoperative days 1, 3, and 7. The largest between-group difference was observed on day 3, followed by a gradual reduction in trismus by day 7 (Figure 1).

Trismus was defined as the change (Δ) in maximal interincisal mouth opening measured on postoperative days 1, 3, and 7. Data are presented as median values with interquartile ranges (IQR) expressed in millimeters (mm).

### 3.6. Osteotomy and Soft Tissue Healing

Soft tissue healing was evaluated using the EHI on postoperative day 7.

No statistically significant differences in EHI scores were observed between the osteotomy and non-osteotomy groups. The median EHI score was4 (IQR: 4–4) in both groups (*p* = 0.465).

## 4. Discussion

### 4.1. Main Findings

The present study evaluated the influence of osteotomy on postoperative morbidity following surgical extraction of impacted mandibular third molars using data derived from a randomized clinical trial.

Procedures requiring osteotomy were associated with significantly longer operative times and increased early postoperative swelling. The most pronounced difference in swelling was observed along facial measurement line A on postoperative day 3.

Trismus demonstrated the most consistent differences between groups, with significantly greater reductions in maximal mouth opening observed in the osteotomy group across all postoperative time points.

In contrast, postoperative pain intensity and early soft tissue healing did not differ significantly between procedures performed with and without osteotomy.

Importantly, multivariable analysis confirmed that osteotomy remained independently associated with increased postoperative swelling (line A, day 3) and greater trismus (day 3) after adjustment for age, sex, and the original six-arm treatment allocation. These findings indicate that bone removal itself contributes to early postoperative morbidity rather than merely reflecting differences in baseline characteristics or treatment allocation.

### 4.2. Comparison with Previous Studies

In the present study, all procedures were performed using a minimally invasive surgical protocol consisting of an envelope flap without vertical releasing incisions, limited osteotomy restricted to the cemento-enamel junction, and controlled internal tooth sectioning using small rotary instruments. Such conservative surgical approaches are commonly recommended to reduce unnecessary surgical trauma during mandibular third molar extraction [12,19,30,31]. The relatively low postoperative pain scores observed in both groups may also reflect the minimally invasive nature of the surgical protocol used in this study, which aimed to limit bone removal and reduce unnecessary tissue trauma.

Previous studies suggest that flap design alone has a limited influence on postoperative morbidity, whereas the overall extent of tissue manipulation and surgical difficulty play a more important role in determining postoperative outcomes [13,30,32].

In our study, procedures requiring osteotomy were associated with significantly longer operative times. Similar observations have been reported in previous investigations indicating that bone removal increases operative duration and surgical complexity during mandibular third molar surgery [13,16,17]. Longer surgical time is generally considered an indirect indicator of greater surgical trauma and tissue manipulation.

The greater surgical trauma associated with osteotomy may also contribute to a more pronounced postoperative inflammatory response. Surgical injury to bone and surrounding tissues triggers the release of inflammatory mediators, including prostaglandins and cytokines, which promote postoperative edema and functional limitation [12,22]. This inflammatory response should not be interpreted solely as a detrimental process, as controlled postoperative inflammation constitutes an integral component of physiological tissue remodeling, with histological evidence demonstrating that mild inflammatory infiltrates may coexist with proper tissue organization and effective healing [33].

Consistent with this mechanism, increased swelling was observed in the osteotomy group. The most pronounced swelling difference occurred along facial measurement line A on postoperative day 3, corresponding to the typical peak of inflammatory edema reported after third molar surgery [12]. This observation corresponds with the typical temporal pattern of postoperative inflammatory edema, which usually reaches its peak approximately 48–72 h after mandibular third molar surgery.

Interestingly, an earlier difference between the groups was observed along facial measurement line C on postoperative day 1. This early change may reflect mechanical displacement of soft tissues and redistribution of postoperative edema rather than the peak inflammatory response. In clinical practice, postoperative swelling after third molar surgery is often described as having two components: an early mechanical displacement of soft tissues immediately after surgery and a later inflammatory edema typically reaching its maximum between 48 and 72 h after the procedure [34,35,36].

Among the evaluated postoperative outcomes, trismus demonstrated the most consistent differences between the groups. Patients undergoing procedures involving osteotomy showed greater reductions in maximal mouth opening during the early postoperative period, suggesting that bone removal substantially contributes to functional limitation after mandibular third molar surgery. Trismus after third molar extraction is generally considered to result from postoperative inflammatory edema and reflex spasm of the masticatory muscles, particularly the masseter and medial pterygoid muscles, caused by surgical trauma and tissue manipulation during the procedure [32,37]. Increased surgical trauma and longer operative time associated with osteotomy may therefore contribute to a more pronounced limitation of mouth opening in the early postoperative period.

Importantly, despite the increased surgical trauma associated with osteotomy, no differences in early soft tissue healing were observed between the groups. Similar observations have been reported in previous studies indicating that bone removal may increase early postoperative morbidity but does not necessarily impair biological wound healing following third molar extraction [20,38,39].

Another relevant aspect of the present study is that prophylactic antibiotics were not routinely administered before or after surgery. Current evidence suggests that routine antibiotic use in healthy patients undergoing third molar extraction has limited influence on postoperative swelling or trismus, as these symptoms primarily reflect the inflammatory response associated with surgical trauma rather than bacterial infection [4,17,22]. Therefore, the absence of antibiotic prophylaxis in the present protocol is unlikely to have influenced the observed postoperative outcomes.

### 4.3. Clinical Implications

The findings of the present study have several practical implications for clinical practice. Procedures requiring osteotomy during mandibular third molar extraction were associated with greater early postoperative morbidity, particularly in terms of swelling and trismus. Clinicians should therefore anticipate a more pronounced early postoperative response in cases where bone removal is necessary.

From a clinical perspective, patients undergoing extractions involving osteotomy may benefit from careful postoperative management aimed at minimizing inflammation and functional limitation. Appropriate patient counseling, clear postoperative instructions, and the use of anti-inflammatory strategies may help facilitate recovery during the early healing period.

Importantly, despite the greater early postoperative morbidity associated with osteotomy, no differences in early soft tissue healing were observed between the groups. This finding suggests that although osteotomy increases surgical trauma, it does not compromise the biological healing of the extraction site. This observation is consistent with previous evidence indicating that local adjunctive interventions after tooth extraction may influence postoperative symptoms, while early soft tissue healing may remain relatively preserved despite differences in surgical management [40].

Overall, the results indicate that osteotomy mainly influences the intensity of early postoperative symptoms rather than the healing capacity of the surgical site. Therefore, the need for osteotomy during third molar surgery may serve as a useful clinical indicator of increased surgical difficulty and the potential for greater early postoperative morbidity.

### 4.4. Limitations

Several limitations of the present study should be acknowledged. First, the analysis represents a secondary evaluation of data derived from a previously conducted randomized clinical trial, and the requirement for osteotomy was determined intraoperatively rather than by random allocation. Consequently, the comparison between procedures with and without osteotomy was not based on random assignment. Although multivariable adjustment was performed for age, sex, and the original six-arm treatment allocation, residual confounding related to surgical difficulty cannot be fully excluded.

Second, the study population consisted primarily of generally healthy young adults, which may limit the generalizability of the findings to older patients or individuals with systemic conditions.

Finally, facial swelling was assessed using line measurements between predefined anatomic landmarks. Although this method is widely used in clinical studies evaluating postoperative swelling after third molar surgery, it may be subject to minor measurement variability.

Despite these limitations, the use of a standardized surgical protocol and prospectively collected clinical data provides a reliable basis for evaluating the relationship between osteotomy and postoperative morbidity following mandibular third molar extraction.

## 5. Conclusions

The requirement for osteotomy during mandibular third molar extraction may be independently associated with greater early postoperative morbidity, including longer surgical duration, increased swelling, and greater trismus. However, osteotomy was not associated with postoperative pain intensity or early soft tissue healing. These findings suggest that although bone removal may increase early postoperative symptoms, it does not appear to compromise the biological healing of the extraction site within the limitations of this secondary analysis.

## Figures and Tables

**Table 1:** Baseline characteristics and surgical duration according to osteotomy status.

Variable	No Osteotomy (*n* = 49)	Osteotomy (*n* = 73)	*p*-Value
Baseline characteristics			
Age, years, median (IQR)	28 (26–29)	30 (27–32)	0.013
Sex, female, *n* (%)	41 (83.7)	55 (75.3)	0.368
Sex, male, *n* (%)	8 (16.3)	18 (24.7)	—
Original six-arm RCT treatment allocation, *n* (%)			
Control	5 (10.2)	17 (23.3)	
PBM	9 (18.4)	11 (15.1)	
A-PRF+	10 (20.4)	9 (12.3)	
CGF	6 (12.2)	14 (19.2)	
A-PRF+ + PBM	9 (18.4)	13 (17.8)	
CGF + PBM	10 (20.4)	9 (12.3)	0.278
Surgical characteristics			
Surgical duration (min), median (IQR)	21 (15–27)	37 (30–45)	<0.001

**Table 2:** Postoperative pain according to osteotomy.

Outcome	Osteotomy Median (IQR)	No Osteotomy Median (IQR)	*p*-Value
Pain, day 1	1 (0–3)	1 (0–2)	0.138
Pain, day 3	1 (0–3)	1 (0–2)	0.138
Pain, day 7	1 (0–3)	0 (0–2)	0.055

Comparison of postoperative pain intensity between osteotomy and non-osteotomy groups. Pain was assessed using an 11-point numeric rating scale (NRS; 0 = no pain, 10 = worst imaginable pain) during follow-up visits on postoperative days 1, 3, and 7. Values are presented as medians with interquartile ranges (IQR). Differences between groups were analyzed using the Mann–Whitney U test.

**Table 3:** Postoperative swelling (line A) relative to baseline according to osteotomy.

Outcome	Osteotomy Median (IQR)	No Osteotomy Median (IQR)	*p*-Value
Δ swellingline A day 1	0 (0–5)	0 (0–5)	0.690
Δ swellingline A day 3	5 (0–8)	0 (−5–5)	0.017
Δ swellingline A day 7	0 (0–0)	0 (0–3)	0.422

Comparison of postoperative facial swelling measured along anatomical line A (lateral canthus–gonion) between osteotomy and non-osteotomy groups. Swelling values represent the change (Δ) between postoperative measurements and baseline values obtained before surgery and are expressed in millimeters (mm). Values are presented as medians with interquartile ranges (IQR). Differences between groups were analyzed using the Mann–Whitney U test.

**Table 4:** Postoperative trismus relative to baseline according to osteotomy.

Outcome	Osteotomy Median (IQR)	No Osteotomy Median (IQR)	*p*-Value
Δ trismus day 1	13 (5–21)	6 (2–18)	0.025
Δ trismus day 3	13 (7–20)	7 (2–16)	0.004
Δ trismus day 7	6 (2–14)	2 (0–8)	0.001

Comparison of postoperative trismus between osteotomy and non-osteotomy groups. Trismus was calculated as the reduction (Δ) in maximal interincisal mouth opening relative to baseline measurements obtained before surgery and is expressed in millimeters (mm). Values are presented as medians with interquartile ranges (IQR). Differences between groups were analyzed using the Mann–Whitney U test.

**Table 5:** Multivariable analysis of factors associated with postoperative outcomes.

Outcome	Variable	β	95% CI	*p*-Value
Swelling day 3 (line A)	Osteotomy	3.44	0.71–6.16	0.014
Trismus day 3	Osteotomy	4.95	1.22–8.68	0.010
Trismus day 3	Male sex	−5.19	−9.67 to −0.71	0.024

Multivariable linear regression models adjusted for age, sex, and the original six-arm treatment allocation from the parent randomized clinical trial. β—regression coefficient; CI—confidence interval.

## Data Availability

The data presented in this study are available on request from the corresponding author.

## Supplementary Materials

The following supporting information can be downloaded at: https://www.mdpi.com/article/10.3390/jcm15103756/s1, Table S1: Postoperative swelling changes relative to baseline according to osteotomy.
