# Supplementary material for: Osteotomy as an Intraoperative Determinant of Early Postoperative Outcomes After Mandibular Third Molar Extraction: A Secondary Analysis of a Randomized Clinical Trial
Source: J Clin Med. 2026 May 13;15(10):3756. doi: 10.3390/jcm15103756 (PMC13206957; doi:10.3390/jcm15103756)
Supplement: Supplementary file 1 [file jcm-15-03756-s001.zip › jcm-4284550-supplementary.pdf]

Supplementary Table S1. Postoperative swelling changes relative to baseline according to osteotomy

Outcome	Osteotomy median (IQR)	No osteotomy median (IQR)	p-value
Δ swelling line A day 1	0 (0–5)	0 (0–5)	0.690
Δ swelling line A day 3	5 (0–8)	0 (-5–5)	0.017
Δ swelling line A day 7	0 (0–0)	0 (0–3)	0.422
Δ swelling line B day 1	4 (0–5)	3 (0–5)	0.260
Δ swelling line B day 3	3 (0–7)	0 (-4–5)	0.054
Δ swelling line B day 7	0 (0–2)	0 (0–2)	0.807
Δ swelling line C day 1	4 (2–5)	3 (0–5)	0.043
Δ swelling line C day 3	5 (0–8)	3 (0–7)	0.229
Δ swelling line C day 7	0 (0–3)	0 (0–1)	0.372

Facial swelling was assessed using linear measurements between predefined anatomical landmarks and expressed as the change (Δ) relative to baseline measurements obtained before surgery. Measurements were performed along three anatomical lines: line A (lateral canthus–gonion), line B (tragus–labial commissure), and line C (tragus–pogonion). Values are expressed in millimeters (mm) as medians with interquartile ranges (IQR).
